# Serial vs. Integrated Outdoor Combined Training Programs for Health Promotion in Middle-Aged Males

**DOI:** 10.3390/sports10080122

**Published:** 2022-08-12

**Authors:** Gerasimos V. Grivas, Konstantina Karatrantou, Athanasios Chasialis, Christos Batatolis, Panagiotis Ioakimidis, Vassilis Gerodimos

**Affiliations:** Department of Physical Education and Sport Science, University of Thessaly, 42100 Trikala, Greece

**Keywords:** concurrent exercise, functional capacity, physical fitness, detraining effect, enjoyment, obesity

## Abstract

The purpose of this study was to examine and compare the training and detraining effects of outdoor serial and integrated combined exercise programs on health, functional capacity, and physical fitness indices. Fifty-one untrained overweight/obese males (47 ± 4 years) were divided into a serial combined (SCG), an integrated combined (ICG), or a control (CG) group. The SCG and ICG implemented a 3-month training (3 sessions/week) consisting of walking and body weight exercises. The only difference between SCG and ICG was the sequence of aerobic and strength training. In SCG, the strength training was performed before aerobic training, while in ICG the aerobic and the strength training were alternated repeatedly in a predetermined order. Health, functional capacity, and physical fitness indices were measured before the training, following the termination of programs, and 1-month after training cessation. Following the training, both the SCG and ICG groups showed reduced blood pressure, heart rate, body fat, and waist-to-hip ratio (3–11%; *p* < 0.001), with improved respiratory function, muscle strength, aerobic capacity, flexibility, and balance (14–61%; *p* < 0.001). After 1-month of training cessation, significant reductions (*p* < 0.05) were observed in health indices and physical fitness without returning to baseline levels. However, there were no differences between SCG and ICG after training and training cessation (*p* > 0.05). In CG, all the above variables did not change. Furthermore, a great percentage of participants in both exercise groups (90%) reported high levels of enjoyment. In conclusion, both serial and integrated outdoor combined walking and body weight strength training programs are enjoyable and equally effective for improving health, functional capacity, and physical fitness indices in overweight/obese middle-aged males.

## 1. Introduction

Physical inactivity is a risk factor for cardiovascular and other chronic diseases, including obesity, diabetes, hypertension, and cancer [1]. In untrained healthy adults, the most recent guidelines for health promotion recommend regular physical activity 3 to 5 times weekly [1]. Compared with other physical activities, walking is a popular, convenient, and free form of aerobic exercise that can be incorporated into everyday life and sustained into old age [2]. Many studies are showing the beneficial effects of walking (indoor or outdoor) on aerobic capacity, lipid profile, body composition, and blood pressure [3,4,5]. In addition to aerobic activities such as walking, strength training could be incorporated into the training program to optimize health-related benefits [6]. For this reason, over the last decades, several researchers recommend the design and implementation of different combined training programs consisting of cardiorespiratory and neuromuscular exercises aiming to improve overall health, functional capacity, and physical fitness parameters [1,6].

A combined training program could be designed and implemented using two different forms: a serial or an integrated form [7,8,9,10]. Serial combined training refers to sessions in which strength training is completed before aerobic exercises (or vice versa); while integrated combined training refers to sessions in which strength and aerobic exercises are alternated repeatedly during each training session [7,8,9,10]. Although numerous studies examined the effects of serial combined aerobic (using walking) and strength training programs on indices of physical fitness and health [11,12,13,14,15]; there is limited information regarding the integrated combined training programs. An integrated combined training program consisting of walking and neuromuscular exercises, called a “fitness trail”, “trim trail” or “parcourse”, could be implemented in different outdoor areas such as forests, transportation rights-of-way, parks, or urban settings aiming to improve overall physical fitness [16]. The few studies that examined the efficiency of “parcourse” exercise programs aimed to improve single or specific indices of health and physical fitness, reporting reduction of body fat, improvement of cardiorespiratory fitness [17,18], and positive effect on participants’ psychological well-being [19]. Thus, the efficacy of integrated combined training programs should be further investigated to have a more “complete picture” of their efficiency.

Furthermore, to our knowledge, no previous study has directly compared the efficiency of serial and integrated combined aerobic (using walking) and strength training (body weight exercises) in middle-aged untrained men. Only two studies [7,8,9,10] directly compared the effectiveness of serial vs. integrated combined training programs reported equivocal findings. Karatrantou et al. [10] showed that serial and integrated combined aerobic (aerobic dance) and strength training (body weight exercises) led to similar benefits in middle-aged females; while Davis et al. [7,8,9] reported that integrated combined aerobic (treadmill running) and strength training (resistance exercises) was slightly superior in improving some of the tested fitness parameters in young collegiate athletes. However, there is evidence that subjects’ characteristics (e.g., age, sex) and physical fitness level are related to different cardiovascular responses, peripheral fatigue development, and substrate utilization during exercise [20,21,22], leading potentially to different neuromuscular and aerobic training adaptations [23,24]. The mode of aerobic exercise, during a combined training program, may be an additional factor that could affect the degree of neuromuscular adaptations [25].

Although regular physical activity is associated with significant health improvements, training cessation will eventually lead to detraining, which has been defined as a partial or complete loss of training adaptations [26]. A previous study suggested that 4 weeks of combined training cessation leads to significant reductions in maximal oxygen uptake (5 to 15%) and maximal force (7 to 10%) [27]. To the best of our knowledge, no study has compared the effects of training cessation on different health and fitness parameters after serial and integrated combined exercise programs.

Therefore, the main objectives of this study were: (a) to examine and directly compared the efficacy of serial and integrated outdoor combined training programs (including walking and body weight strength exercises) on health, functional capacity, and physical fitness indices, and (b) the retention of performance gains 1 month after the completion of the training regimens in middle-aged overweight/obese males. We have also examined the participants’ enjoyment following the serial and the integrated combined training programs.

## 2. Materials and Methods

### 2.1. Participants

The study participants were 51 healthy untrained overweight (BMI ≥ 25)-obese (BMI ≥ 30) men aged between 42 and 54 years old. From the total sample, 35 were overweight and 16 were obese. Before the study, participants’ health (health history questionnaire, resting electrocardiogram, and echocardiogram examined by a cardiologist) and activity status were assessed. All participants (1) were inactive (i.e., without systematic strength or endurance training for at least one year before the study), (2) were free from chronic illnesses, and (3) had no injuries or diseases. Participants were informed about the risks, discomforts, and benefits associated with the study. Thereafter, all participants received detailed information about the study design, measurements, and procedures and were required to give a written informed consent form. The procedures followed the Helsinki declaration of 1975, as revised in 2000, and approval was received from the Ethics Committee of the local university.

### 2.2. Study Design

The effects of serial and integrated outdoor combined training programs on health, functional capacity, and physical fitness indices were examined using a randomized controlled design. A computer-generated list of random numbers was used for the allocation of the participants to one of the three groups (17 participants per group). The main investigator and the outcome assessors were blinded for the allocated intervention during the entire period of data collection. During the study, participants were requested not to discuss their intervention with the main investigator and the outcome assessors.

Two weeks before the beginning of the training protocols, subjects completed a laboratory familiarization session and were informed about the proper form of exercises. Thereafter, indices of health, overall fitness, and functional capacity were assessed on 3 separate days. Two days following the pre-training testing, the subjects were randomly divided into three equal groups (*n* = 17 per group): serial combined group (SCG; 12 overweight and 5 obese men), integrated combined group (ICG; 12 overweight and 5 obese men), or control group (CG; 11 overweight and 6 obese men) (Table 1). During the study, SCG and ICG participants were involved in a 3-month outdoor combined aerobic (using walking) and strength (using body weight exercises) training program while the CG simply maintained their normal daily activities. After the 3-month training program, SCG and ICG participants completed an enjoyment questionnaire.

All measurements were repeated after 3 months of training and 1 month after training cessation. The measurements were made at the same time of the day to minimize the effects of circadian fluctuation and started two days after the end of the training intervention. Before testing, all participants were asked to refrain from: (a) alcohol or caffeine consumption within 3 h of testing, (b) smoking within 3 h of testing, and (c) strenuous physical strength and endurance activities within 48 h of testing.

### 2.3. Training Programs

Both groups (SCG and ICG) participated in a 3-month combined outdoor training program (3 times per week). The duration of each training session was 58 to 88 min and consisted of 15 min of warm-up (10 min of walking and 5 min of stretching exercises), followed by 33 to 63 min of serial or integrated combined training. Each session was completed with a 10-min cool-down period (5 min of walking and 5 min of stretching exercises). Both groups performed the same aerobic (walking) and strength exercises (body weight exercises) during the workouts, using equivalent intensity, duration, volume, and training frequency (Table 2). Training intensity was controlled by the HR monitor (Polar RS400) during the training session. The mean heart rate was not different between the two exercise groups during the 3-month training intervention (69 ± 5% of HR_max_ in the SCG vs. 70 ± 6% of HR_max_ in the ICG). Thus, the only difference between the two groups was the sequence of aerobic and strength training. In SCG, the strength training was performed before aerobic training, while in ICG the aerobic and the strength training were alternated repeatedly in a predetermined order (3 min of walking/2 min of strength training).

The aerobic training program, for both exercise groups, consisted of brisk walking with dynamic arm moves forward and backward to increase participants’ heart rate and maximize health benefits at an intensity corresponding to 65–80% of age-predicted HR_max_ (Polar Electro, Kempele, Finland) for 21 to 39 min. The intensity and duration of training progressively increased during the training program (see Table 2) according to the procedures described previously [6]. The strength training program consisted of 5 body weight exercises for all major muscle groups (sit-ups, dorsals, push-ups, tricep dips, and lunges). The number of repetitions (8–12 RM) and sets (2–4) of the body weight strength exercises progressively increased during the training program (see Table 2) according to the procedures described previously [6]. All training sessions were supervised by the project staff to ensure completion and adherence to the training program.

### 2.4. Testing Procedures

Before and after the completion of the 3-month training period and 1 month following the termination of the program different health, functional capacity, and physical fitness indices were measured. Before functional capacity and physical fitness testing, the participants performed a standardized 15 min warm-up (10 min of stationary cycling, 5 min of static and dynamic stretching exercises).

### 2.5. Health Indices

Body height and body mass were measured using a physical beam scale (Seca model 220, Seca, Hamburg, Germany), and body composition was measured using bioelectrical impedance analysis (Maltron 900) [1]. Waist to hip ratio using ergonomic circumference measuring tape (Seca 203), blood pressure using an electronic blood pressure monitor (A&D-UA-851) and respiratory function (forced vital capacity-FVC, forced expiratory volume in 1s-FEV_1_) using a portable spirometer (Micro Medical Micro) were also measured [1].

### 2.6. Functional Capacity-Physical Fitness Indices

Lower back and hamstring flexibility was assessed with the sit-and-reach test using a Flex-Tester box (Novel Products Inc., Rockton, IL, USA) [1]. Static balance was evaluated, on both legs, using the single-leg stance test [28] and dynamic balance was evaluated using the timed up and go test (TUG) [29].

Strength and power: Muscular endurance of abdominal muscles, chest and biceps muscles, triceps muscles as well as trunk extensors muscles, were assessed using the “curl-up test,” the modified “knee push-up test”, the “dip test,” [1], and “Ito test” [30], respectively. Also, vertical jumping performance was assessed using the squat jump test (SJ) with an Optojump system (Microgate, Bolzano, Italy), as previously described by Tsourlou et al. [31].

Cardiorespiratory fitness: Cardiorespiratory fitness was assessed using the Ebbeling submaximal treadmill protocol [32]. At the end of each stage, the RPE was obtained using the 20-point Borg scale. Participants’ heart rate was measured after each stage and in the first minute following the termination of the walking test. Finally, VO_2max_ was also estimated using the following equation proposed by Ebbeling: VO_2max_ = 15.1 + 21.8 (speed in mph) − 0.327 (heart rate in bpm at 5% grade) − 0.263 (speed × age in years) + 0.00504 (heart rate in bpm at 5% grade × age in years) + 5.98 (sex: female = 0, male = 1) [32].

### 2.7. Enjoyment

Participants’ enjoyment following the exercise program was assessed with a 4-item scale (e.g., “I enjoyed this exercise program very much”) based on the Intrinsic Motivation Inventory [33]. All items were scored on a 5-point Likert scale from 1 (strongly disagree) to 5 (strongly agree).

### 2.8. Statistical Analysis

Results are presented as means ± standard deviations. Data were analyzed with IBM SPSS Statistics v.23 software (IBM Corporation, Armonk, NY, USA). The normality of data was examined using the Shapiro–-Wilk test. 2-way ANOVAs (group × time; 3 × 3) with repeated measures on the “time” factor were used to analyze the data. Sidak pairwise comparisons were applied to locate the significantly different means within and between groups. One-way ANOVAs were used between groups to compare the relative changes from pre- to post-training, from pre- to training cessation, and from post- to training cessation in fitness and health indices. The magnitude of the difference was assessed by Hedges’ g (g), and was considered small (0.2 < g ≤ 0.5), moderate (0.5 < g ≤ 0.8), or large (g > 0.8). Statistical significance was set at *p* < 0.05 for all analyses.

## 3. Results

### 3.1. Health Indices

ANOVAs showed significant “group × time” interaction effects on health indices (*p* < 0.001, Table 3). Compared to pre-training values, after 3 months of training (post-training) both groups (SCG and ICG) improved on all health indices (*p* < 0.001, g = 0.4–2.5). More specifically, we found a moderate decrease in %BF, a small decrease in waist-to-hip ratio, and a moderate-large decrease in arterial blood pressure as well as a large increase in FVC and FEV_1_. There were no differences between SCG and ICG. In CG, the above variables did not change (*p* > 0.05). The changes from pre-training to post-training for all health indices were greater in SCG and ICG vs. CG (*p* < 0.001). After 1 month of training cessation, all health indices were still higher compared to the pre-training values in SCG and ICG (*p* < 0.001, g = 0.2–1.5), but significantly decreased compared to the post-training values (*p* < 0.05, g = 0.2–0.8). There was no difference between SCG and ICG after training cessation (*p* > 0.05). CG did not change after training cessation (*p* > 0.05).

### 3.2. Physical Fitness and Functional Capacity Indices

ANOVAs showed significant “group × time” interaction effects on SJ performance, endurance strength (*p* < 0.001, Table 4), cardiorespiratory fitness (*p* < 0.001, Table 5), as well as on flexibility, and balance (*p* < 0.001, Table 6). After 3 months of training (post-training), both training groups (SCG and ICG) increased SJ performance, muscle endurance of the upper body, flexibility, static balance, and VO_2max_ estimation), while showing decrease heart rate, RPE, and time to complete the TUG test (*p* < 0.01; g = 0.7–8.6). On the other hand, in CG, the above physical fitness and functional capacity indices did not change. There was no difference between SCG and ICG. Comparisons between groups revealed that SJ performance, upper body muscle endurance, flexibility, static balance, and VO_2max_ estimation values were greater in SCG and ICG vs. CG (*p* < 0.05). Heart rate, RPE, and the time to complete the TUG test were lower in the two training groups vs. CG (*p* < 0.01). After 1 month of training cessation, SJ performance, muscle endurance of the upper body, flexibility, static balance, and VO_2max_ estimation were still higher compared to the pre-training values in SCG and ICG (*p* < 0.01), but decreased compared to the post-training values (*p* < 0.05). Heart rate and the time to complete the TUG test were still lower compared to the pre-training values in SCG and ICG (*p* < 0.01), but increased compared to the post-training values (*p* < 0.05).

### 3.3. Enjoyment

According to the results of the study, a great percentage (90%) of both groups reported high levels of enjoyment and satisfaction during the combined exercise programs, as they reported high scores on the 5-point scale of the questionnaire. There was no difference in the level of enjoyment between SCG (total score from all questions: 4.4 ± 0.3) and ICG (total score from all questions: 4.4 ± 0.2).

## 4. Discussion

The objective of this study was to examine and compare the effects of serial and integrated outdoor combined training programs (including walking and body weight strength exercises) and verify the effects of subsequent training cessation on health, functional capacity, and physical fitness indices in untrained overweight/obese middle-aged males. The main findings of the present study were that both training regimens decreased body fat, body waist-to-hip ratio, and blood pressure, while increasing respiratory function and improving overall fitness and functional capacity. Conversely, after 1 month of training cessation, both groups had significant reductions in health indices, functional capacity, and physical fitness without returning to the baseline levels.

Our findings for the serial combined aerobic (using walking) and strength training (using body weight strength exercises) program are in line with findings from previous studies reporting improvements in aerobic capacity, muscle strength, body fat, and body weight in middle-aged men [11,12,13,14,15] after serial combined programs. Others, however, did not observe positive training adaptations in body weight, BMI, and waist circumference [13,14,15]. The differences observed between results from earlier studies and the present study could be attributed to different subjects’ characteristics, and training load, but mainly to the order of exercises, which may all amplify the interference effect and reduce the efficacy of serial combined strength and aerobic training programs in improving neuromuscular performance [34,35]. Strength and endurance training cause significantly different or even opposite adaptations. Strength training causes skeletal muscle hypertrophy, by activating the mammalian/mechanistic target of the rapamycin (mTOR) signaling pathway, and neuromuscular responses [36,37], while aerobic training causes skeletal muscle oxidative and metabolic capacity to increase [38] by activating adenosine monophosphate (AMP)-activated protein kinase (AMPK). There is also evidence that AMPK interferes with mTOR signaling via tuberous sclerosis complex 2 (TSC2), suppressing protein synthesis [39]. Taking all the above into consideration, the combination of those two different modes of training in the same period may lead to the so-called “interference effect” [40]. The interference effect is the phenomenon by which adaptation to combined strength and aerobic training is diminished compared to separately training only strength or endurance [40]. For example, several studies reported that residual fatigue caused by prior aerobic training reduces the neural input to the exercised muscle (during aerobic training) resulting in decrements in force output and the rate of force development, as well as attenuation of neuromuscular adaptations [41]. On the other hand, other studies revealed that the order of exercises during combined training does not affect neuromuscular responses [25]. The efficacy of serial combined training programs and the presence of an interference effect can be affected by several factors such as the type of exercise, the training level, the muscle groups involved, as well as the inter-individual variations [25,34,35,41]. It is possible that the relatively low level of physical fitness of the untrained middle-aged males in our study, in conjunction with the exercise mode and the order of exercises during the serial combined training program, attenuated the interference effect and promoted the neuromuscular adaptations in our study.

Furthermore, only a few studies have examined the effects of integrated combined programs in middle-aged men. Previous studies showed similar improvements in aerobic capacity, muscle strength, and endurance in middle-aged men [42,43,44]. However, the same previous studies have found no improvements in body fat, body weight, and waist circumference [42,43]. The possible explanations for these discrepancies could be related to the different aerobic training modalities and the differences in subjects’ characteristics, loading parameters, and frequency of training.

There is evidence that the positive effects on physical fitness that occur after training disappear if the stimulus is interrupted [26,27]. Thus far, data on the effects of combined (serial or integrated) training cessation remain scarce. Our findings are similar to previous studies reporting that after 3–12 weeks of serial combined training, the cessation of training was followed by a reduction of aerobic capacity, muscle endurance strength, balance, and flexibility in young and elderly individuals, without returning to the baseline levels [27,45]. On the other hand, some studies have found no changes in aerobic capacity, body weight, body fat, BMI, and static balance [45,46,47]. The conflicting results from our study compared to these studies may be due to the different subjects’ characteristics, the different aerobic training modalities, the different loading parameters such as frequency, volume, and the lower duration of training cessation. Unfortunately, no previous study has evaluated the effects of training cessation after integrated combined programs, so comparison is not possible.

Concerning the difference between serial and integrated combined training, there are only two studies that directly compared the effectiveness of two combined training types and reported conflicting results [7,8,9,10]. In the study of Karatrantou et al. [10], including untrained middle-aged females, serial and integrated combined training consisted of aerobic dance and calisthenics conferred analogous adaptations in all measured parameters. In contrast, the study of Davis et al. [7,8,9] was performed on young athletes and used combined running and strength training. The authors reported that, for most variables (cardiorespiratory fitness, muscle endurance of the lower body, and flexibility of the upper body), the integrated combined training led to better results compared to the serial training. The results of Davis et al. differ from those of the present study and the study by Karatrantou et al. [10]. The discrepancy in the findings may be related to the different ages, sex, and methods implemented in the training program. According to the study by Davis et al. [7,8,9], the greater results in most variables for integrated combined training compared to serial combined training were due to the much higher heart rate during resistance training in the integrated combined training (65% vs. 32% of heart rate reserve). It had also been observed that in integrated combined training the increases in heart rate during resistance training cause a reduction of delayed onset muscle soreness and accelerate muscle recovery [7,8,9].

Although it is well known that regular participation in physical activities is associated with significant health improvements, many participants drop out of exercise programs. To avoid the dropout of participants, one important strategy is the promotion of positive feelings and satisfaction during the exercise program [48]. Our results in both exercise groups are in agreement with the studies of Wilke et al. [49] and Heinrich, Patel, O’Neal and Heinrich [50], showing high levels of enjoyment following a combined exercise program in adults. An additional important advantage of the present study is the outdoor exercise in the natural environment. There is evidence that outdoor exercise causes significant improvements in mental well-being, increases ratings of mood [51], and reduces tiredness and stress [52].

In conclusion, a well-designed serial and integrated combined 3-month exercise program (including walking and body weight exercises) can produce positive adaptations in middle-aged overweight/obese males and it seems that these benefits are still observed after 1 month of training cessation. The choice of a combined training program (serial or integrated) is not important, considering that both programs improved health and physical fitness indices in middle-aged overweight/obese males. However, further studies are needed to compare both training programs with a longer training duration.

## Figures and Tables

**Table 1:** Subjects characteristics per group (values are means ± SD).

Variables	SCG (*n* = 17)	ICG (*n* = 17)	CG (*n* = 17)
Age (years)	46 ± 4	46 ± 3	47 ± 3
Body mass (kg)	95 ± 19	92 ± 15	94 ± 16
Body height (m)	1.78 ± 0.56	1.76 ± 0.50	1.78 ± 0.44
BMI (kg/m^2^)	29.6 ± 5	29.2 ± 4	29.3 ± 4

SCG: serial combined group; ICG: integrated combined group; CG: control group; BMI: body mass index.

**Table 2:** Gradual increase of loading parameters during the serial and integrated combined training programs.

	Weeks			
	1–3	4–5	6–8	9–12
**Serial Combined Training**				
***Aerobic training (walking)***				
Intensity (% HR_max_)	65–72%	70–75%	70–80%	75–80%
Duration (min)	21	30	30	39
***Strength training (body weight exercises)***				
Sets	2	3	3	4
Reps/set	8–10	10	12	12
Rest time/set (s)	40–60	40–60	40–60	40–60
**Integrated Combined Training**				
***Aerobic training (walking)***				
Intensity (% HR_max_)	64–73%	70–76%	70–80%	74–80%
Duration (set × time)	7 × 3 min	10 × 3 min	10 × 3 min	13 × 3 min
***Strength training (body weight exercises)***				
Sets	2	3	3	4
Reps/set	8–10	10	12	12
Rest time/set (s)	Non-applicable			

% HR_max_: percentage of the age-predicted maximum heart rate as recorded during the training program by the Polar RS400 heart rate monitor.

**Table 3:** Health indices in the three groups (SCG, ICG, and CG) pre-training, post-training, and after training cessation (values are means ± SD).

Variables	Groups	Pre-Training	Post-Training	Training Cessation
**Body fat (%)**	SCG	30 ± 4	27 ± 3 *†	29 ± 3 *#†
	ICG	30 ± 4	27 ± 4 *†	29 ± 4 *#†
	CG	31 ± 3	31 ± 3	31 ± 3
**Waist-to-hip ratio**	SCG	0.98 ± 0.05	0.96 ± 0.04 *	0.97 ± 0.04 *#
	ICG	0.98 ± 0.01	0.96 ± 0.01 *	0.97 ± 0.01 *#
	CG	0.97 ± 0.01	0.97 ± 0.01	0.97 ± 0.01
**Systolic blood pressure (mmHg)**	SCG	121 ± 7	117 ± 6 *†	120 ± 6 *#†
	ICG	123 ± 5	118 ± 4 *†	120 ± 3 *#†
	CG	125 ± 3	127 ± 3	126 ± 3
**Diastolic blood pressure (mmHg)**	SCG	79 ± 6	75 ± 5 *†	77 ± 5 *#†
	ICG	78 ± 2	74 ± 3 *†	77 ± 3 *#†
	CG	80 ± 2	80 ± 2	80 ± 2
**FVC (L)**	SCG	4.59 ± 0.17	4.75 ± 0.20 *†	4.70 ± 0.19 *#†
	ICG	4.52 ± 0.06	4.67 ± 0.06 *†	4.62 ± 0.07 *#†
	CG	4.25 ± 0.33	4.23 ± 0.36	4.24 ± 0.36
**FEV_1_ (L)**	SCG	3.90 ± 0.10	4.03 ± 0.11 *†	3.98 ± 0.10 *#†
	ICG	3.84 ± 0.07	3.96 ± 0.07 *†	3.93 ± 0.06 *#†
	CG	3.75 ± 0.15	3.73 ± 0.18	3.76 ± 0.17

SCG: serial combined group; ICG: integrated combined group; CG: control group; FVC: forced vital capacity; FEV_1_: forced expiratory volume in 1 s; * *p* < 0.001 pre-training vs. post-training and pre-training vs. training cessation in SCG and ICG, # *p* < 0.05 post-training vs. training cessation in SCG and ICG, † *p* < 0.001 vs. CG.

**Table 4:** Muscular performance in the three groups (SCG, ICG, and CG) pre-training, post-training, and after training cessation (values are means ± SD).

Variables	Group	Pre-Training	Post-Training	Training Cessation
**Squat jump (cm)**	SCG	20 ± 1	21 ± 1 *†	21 ± 1 *#†
	ICG	19 ± 1	20 ± 1 *†	20 ± 1 *#†
	CG	20 ± 1	19 ± 1	19 ± 1
**Sit-ups** **(reps)**	SCG	33 ± 3	54 ± 3 *†	46 ± 3 *#†
	ICG	34 ± 3	53 ± 4 *†	45 ± 4 *#†
	CG	32 ± 4	33 ± 4	34 ± 4
**Ito test (s** **)**	SCG	70 ± 6	183 ± 5 *†	153 ± 6 *#†
	ICG	71 ± 4	179 ± 7 *†	150 ± 6 *#†
	CG	69 ± 4	68 ± 5	70 ± 5
**Push-ups (reps)**	SCG	25 ± 2	37 ± 2 *†	31 ± 2 *#†
	ICG	26 ± 2	37 ± 3 *†	31 ± 3 *#†
	CG	25 ± 2	25 ± 3	26 ± 3
**Dip test (reps)**	SCG	15 ± 1	29 ± 2 *†	24 ± 2 *#†
	ICG	15 ± 3	26 ± 4 *†	22 ± 3 *#†
	CG	15 ± 2	15 ± 2	16 ± 3

SCG: serial combined group; ICG: integrated combined group; CG: control group; * *p* < 0.001 pre-training vs. post-training and pre-training vs. training cessation in SCG and ICG, # *p* < 0.05–0.001 post-training vs. training cessation in SCG and ICG, † *p* < 0.05–0.001 vs. CG.

**Table 5:** Cardiorespiratory fitness in the three groups (SCG, ICG, and CG) pre-training, post-training, and after training cessation (values are means ± SD).

Variables	Groups	Pre-Training	Post-Training	Training Cessation
**HR_test_ (beats/min)**				
Stage 1	SCG	112 ± 5	99 ± 6 *†	104 ± 7 *#†
	ICG	112 ± 6	100 ± 5 *†	105 ± 5 *#†
	CG	116 ± 8	117 ± 8	117 ± 8
Stage 2	SCG	132 ± 8	119 ± 8 *†	123 ± 8 *#†
	ICG	132 ± 6	118 ± 9 *†	122 ± 9 *#†
	CG	135 ± 9	135 ± 9	135 ± 8
Stage 3	SCG	148 ± 3	137 ± 5 *†	141 ± 4 *#†
	ICG	148 ± 3	137 ± 6 *†	142 ± 6 *#†
	CG	150 ± 2	150 ± 2	151 ± 2
**HR_rec_ (beats/min)**				
1st min	SCG	113 ± 12	96 ± 7 *†	102 ± 7 *#†
	ICG	111 ± 6	93 ± 5 *†	99 ± 5 *#†
	CG	114 ± 3	115 ± 3	115 ± 4
**RPE_test_**				
Stage 1	SCG	10 ± 2	8 ± 0.4 *†	9 ± 1 *#†
	ICG	10 ± 0.9	9 ± 0.8 *†	10 ± 0.5 *#†
	CG	11 ± 0.7	12 ± 0.8	11 ± 0.6
Stage 2	SCG	14 ± 1	11 ± 1 *†	13 ± 0.9 *#†
	ICG	14 ± 0.9	11 ± 1 *†	12 ± 0.7 *#†
	CG	14 ± 1	14 ± 0.9	14 ± 1
Stage 3	SCG	17 ± 0.5	15 ± 0.9 *†	16 ± 0.7 *#†
	ICG	17 ± 0.5	14 ± 0.5 *†	16 ± 0.5 *#†
	CG	17 ± 0.5	17 ± 0.5	17 ± 0.5
**VO_2max_ estimation**				
VO_2max_ (mL/kg/min)	SCG	38 ± 2	42 ± 4 *†	40 ± 3 *#†
	ICG	39 ± 1	43 ± 2 *†	41 ± 2 *#†
	CG	38 ± 2	38 ± 2	38 ± 2

SCG: serial combined group; ICG: integrated combined group; CG: control group; HR test: heart rate values during the submaximal exercise; HR_rec_: 1-min recovery heart rate; RPE_test_: rating of perceived exertion during the submaximal exercise; VO_2max_ estimation: was used the following equation: VO_2max_ = 15.1 + 21.8 (speed in mph) − 0.327 (heart rate in bpm) − 0.263 (speed × age in years) + 0.00504 (heart rate in bpm × age in years) + 5.98 (gender: female = 0, male = 1). * *p* < 0.001 pre-training vs. post-training and pre-training vs. training cessation in SCG and ICG, # *p* < 0.01 post-training vs. training cessation in SCG and ICG, † *p* < 0.01 vs. CG.

**Table 6:** Flexibility and balance in the three groups (SCG, ICG, and CG) pre-training, post-training, and after training cessation (values are means ± SD).

Variables	Groups	Pre-Training	Post-Training	Training Cessation
**Sit and reach (cm)**	SCG	14 ± 1	19 ± 1 *†	17 ± 2 *#†
	ICG	13 ± 3	17 ± 3 *†	16 ± 3 *#†
	CG	14 ± 2	14 ± 2	13 ± 2
**Static balance right leg (s)**	SCG	55 ± 3	73 ± 3 *†	71 ± 3 *#†
	ICG	53 ± 4	73 ± 3 *†	71 ± 3 *#†
	CG	54 ± 3	54 ± 3	55 ± 3
**Static balance left leg (s)**	SCG	52 ± 3	72 ± 5 *†	70 ± 4 *#†
	ICG	52 ± 5	71 ± 4 *†	69 ± 4 *#†
	CG	54 ± 2	52 ± 4	53 ± 4
**TUG test (s)**	SCG	4.61 ± 0.23	4.05 ± 0.20 *†	4.22 ± 0.20 *#†
	ICG	4.71 ± 0.14	4.13 ± 0.08 *†	4.25 ± 0.09 *#†
	CG	4.83 ± 0.19	4.80 ± 0.22	4.76 ± 0.21

SCG: serial combined group; ICG: integrated combined group; CG: control group; * *p* < 0.01–*p* < 0.001 pre-training vs. post-training and pre-training vs. after training cessation in SCG and ICG, # *p* < 0.05 post-training vs. after training cessation in SCG and ICG, † *p* < 0.001 vs. CG.

## Data Availability

The data presented in this study are available from the corresponding author on reasonable request.
